# Innate immune response-mediated late increase in SuPAR in multi-trauma patients

**DOI:** 10.1186/cc12224

**Published:** 2013-03-19

**Authors:** K Timmermans, M Kox, M Vaneker, P Pickkers

**Affiliations:** 1 Radboud University Nijmegen Medical Center, Nijmegen, the Netherlands

## Introduction

The soluble form of urokinase-type plasminogen activator (suPAR) has been identified as a marker for immune activation and is demonstrated to accurately predict outcome in patients with sepsis or infectious diseases. In multi-trauma patients a considerable immunological response also occurs that is related to multiple organ failure and patient outcome. We investigated the kinetics of suPAR, correlation with the immune response and outcome in multi-trauma patients.

## Methods

Blood was obtained from adult multi-trauma patients (*n = *63) on arrival at the emergency room (ER) of the Radboud University Nijmegen Medical Centre and days 1, 3, 5, 7, 10 and 14 following trauma. Plasma concentrations of TNFα, IL-6, IL-10, IFNγ, IL-8 and MCP-1 were determined by Luminex, and SuPAR concentrations using ELISA. Clinical data were collected from electronic patient files. Concentrations, areas under the curve (AUC) and regression coefficients were statistically analyzed. Spearman correlation coefficients were calculated and differences between survival/nonsurvival groups were analyzed using unpaired Student *t *tests.

## Results

SuPAR values at admission to the ER were higher in nonsurvivors compared with survivors (*n=*16), mean ± SEM 4.1 ± 0.6 ng/ml vs. *n = *40, 3.0 ± 0.2 ng/ml, *P *= 0.03). SuPAR levels increased in time. An increase of suPAR did not predict or precede death, however. SuPAR AUC from ER to day 5 tended to correlate with injury severity score (*r *= 0.5, *P *= 0.07). Plasma cytokines in the ER did not correlate with suPAR measured at the same time (for example, TNFα: *r *= 0.2, *P *= 0.37, IL-10: *r *= -0.02, *P *= 0.91), while cytokine concentrations at the ER did correlate with suPAR levels at days 3 (TNFα: *r *= 0.6, *P <*0.01, IL-10: *r *= 0.5, *P *= 0.02) and 5 (TNFα: *r *= 0.7, *P <*0.01).

## Conclusion

Plasma concentrations of SuPAR measured at admission to the ER are associated with overall survival of multi-trauma patients. Furthermore, suPAR concentrations increased during hospital admission, with most pronounced increases found in patients that suffered more serious injury and related to the innate immune response determined in the ER. These results indicate that suPAR is an innate immune response-induced late mediator in multi-trauma patients.

